# Setting the standard for machine learning in phase field prediction: a benchmark dataset and baseline metrics

**DOI:** 10.1038/s41597-024-04128-9

**Published:** 2024-11-23

**Authors:** Laura Hannemose Rieger, Klemen Zelič, Igor Mele, Tomaž Katrašnik, Arghya Bhowmik

**Affiliations:** 1 Department of Energy and Conversion Storage, Technical University of Denmark (DTU), Lyngby, 2800 Kgs Denmark; 2 National Institute of Chemistry, Department of Materials Chemistry, Ljubljana, 1000 Slovenia; 3 University of Ljubljana, Faculty of Mechanical Engineering, Ljubljana, SI-1000 Slovenia

**Keywords:** Batteries, Computational methods, Batteries

## Abstract

Phase field models are an important mesoscale method that serves as a bridge between the atomic scale and the macroscale, used for modeling complex phenomena at the microstructure level. Machine learning can be employed to accelerate these simulations, enabling faster and more efficient analyses. However, the development of new machine learning algorithms depends on access to extensive datasets. This work introduces an accessible and well-documented dataset aimed at benchmarking new machine learning algorithms. We validate the dataset with a benchmark using U-Net regression, a widely used neural network architecture. Although direct comparisons are limited by the lack of existing benchmarks, our model’s error metrics are competitive with previous work and generalize across multiple domain sizes. This contribution provides a valuable resource for future efforts in machine learning model development for phase field simulations and demonstrates the potential of U-Net regression, highlighting the scope for novel method development in this area.

## Background & Summary

Material properties inherently encompass multiple time and length scales, ranging from the atomistic to the meso-scale and microstructural levels, and extending to the macroscopic scale. Simulations are critical to the discovery of new materials and establishing quantitative structure-property correlations. The development of simulation tools that combine the traditional physics-based approach with data-driven machine learning models^1^ can accelerate materials design across scales. For example, creating a more robust and integrated framework with an interoperable data infrastructure^2^, physics-based models^3^ and machine learning tools^4^ can revolutionize the discovery of novel energy storage systems^5^. Successful implementation of data-driven machine learning (ML) models is dependent on access to physically valid, curated datasets that can be automatically retrieved and expanded with further simulations. Extensive and validated datasets lead to machine learning models that lay the foundation for accelerated but reliable simulations.

Functioning as a well-established meso-scale modeling method, the phase field method plays a crucial role in bridging the gap between atomistic scale models and those operating on a macro-scale. This is achieved by extracting local free energy density information from lower scale models and incorporating phase field resultant trends as inputs into higher scale models and/or to predict phenomena observed at higher scales. Phase field captures the dynamic behaviors of materials at mesoscale describing microstructural evolution and phase transformation, with solidification and phase separation standing out as common applications^6,7^. This approach has significantly contributed to understanding of diverse processes, ranging from grain growth^8,9^, crack propagation^10^, dendrite growth^11,12^, and dislocation dynamics^13^ to the dynamics of interphase boundaries^14^ and membranes^15^. In battery materials, phase field models are used to predict phase transformations, diffusion, and microstructural evolution during charge-discharge cycles^16^.

Specific results comprise modelling lithiation/de-lithiation dynamics of active electrode particles composed of phase separating materials^14,17^ and overpotentials associated with the movement of the phase boundary between Li-rich and Li-poor regions of it. Resulting intra-particle stresses^18^ significantly influence electrode performance and degradation. Intra-particle transport and resulting chemical potentials significantly influence electrode response under application of direct and alternating currents^19,20^. While phase field methods represent one of the very important methods for computational optimization of materials, its full potential can only be achieved if numerous simulations covering long time-scales can be executed. The current limitation comes from the computational complexity of solving systems of differential equations for each short time step during simulations. This results in limited temporal and spatial resolutions and keeps systematic parametric studies for searching multi-dimensional design space out of reach.

To resolve this challenge, a data driven approach that leverages latest developments in AI accelerated physics simulations can be envisioned^21,22^. Machine learning models, while computationally expensive during training, can predict complete trajectories much faster. However, building them requires high-quality, easy-to-access datasets. We introduce a comprehensive benchmark of phase field simulations across multiple domain sizes. A common challenge in machine learning is the divergence between raw data and the format required by machine learning experts. We publish the raw data as generated, along with the processed dataset and the code used for processing. The code for every step, from data generation and curation to the training and evaluation of a deep neural network (DNN), is available.

Our data generation utilized a customized phase field model based on the Cahn-Hilliard equation, as detailed in prior works^17,20^. To accurately capture spinodal decomposition, we incorporated a spinodal chemical potential shape^23^ into our model. The parameters of the Cahn-Hilliard equation were chosen to correspond to the properties of lithium iron phosphate (LFP) battery electrodes. Consequently, we derived a model for the (de)lithiation process within a single nano-particle of LFP material. By employing this model, we computed relaxation trajectories for the lithium concentration field within LFP nano-particles under various initial conditions and particle sizes. Our systematic parametric investigation resulted in the generation of a large dataset comprising microstructure evolution trajectories for LFP particles during relaxation.

There has been a recent surge in interest towards predicting microstructure evolution with machine learning algorithms such as recurrent neural networks (RNN) or convolutional neural networks (CNN)^24–28^.In contrast to the work by de Oca Zapiain *et al*.^24^ requiring physical simulation to be run during inference, we have taken a different approach to create a baseline model with U-Net architecture. It does not require subsequent simulation steps to be run and the entire trajectory is recovered via machine learning. The model developed by Peivaste *et al*.^28^ is closely related but requires longer simulations of the early stages of the phase field trajectory and datasets with only low-entropy initial configurations.

We have added built-in self-consistency to our machine learning model, enforcing a physically plausible development of the microstructure. This allows us predict future time frames in more complex scenarios because it does not need to learn to ’keep track’ of the correct phase fractions of each phase at each step by ensuring that it is by design consistent from input to prediction. We find that the machine learning model can accurately predict the next step from high-entropy initial configurations where the trajectory develops quickly. Consequently, we create and evaluate our developed machine learning algorithm on a wide range of initial configurations. Since there are currently no publicly available baselines available, we envision that our contribution of a large diverse dataset of microstructure evolutions will serve to provide a benchmark to the field.

Previously, Wheeler *et al*.^29^ presented a list of benchmark problems, including spinodal decomposition, to compare the quality of numerical implementations of phase field models. Complementary to our work, their focus is on benchmarking numerical methods, whereas we present a dataset specifically designed to benchmark machine learning algorithms that can accelerate phase field simulations.

**Fig. 1:**
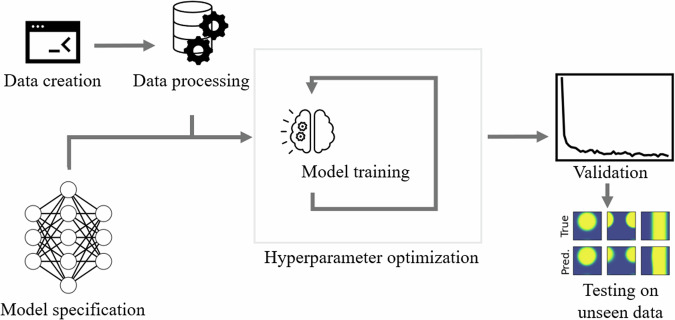
Visualization of the complete workflow.

In summary, our contributions in this paper are as follows: We present an open-source, easy-to-use phase field model based on Cahn-Hilliard equation. The presented model is parameterized for the case of LFP Li-ion battery active material. This model was used for generation of presented novel comprehensive dataset of phase field simulations over a wide range of input parameters. Utilizing this dataset we train a machine learning model and evaluate it on both in-distribution and out-of-distribution data as shown in Fig. 1. We show that the machine learning model can accurately predict the future phase field trajectory. Using this model, we show the need for a standardized and openly available dataset by demonstrating the dependency of predictive accuracy on parameters such as domain size and starting point. Finally, we contribute the code that, due to its modular nature, can be used to quickly try out new machine learning algorithms or the training on new data, allowing for the acceleration of future machine learning model development and materials science research.

## Methods

This section first briefly introduces the modelling framework of the phase field model, which was written in a structured way using computationally efficient C code to enable further extensions of the model, followed by an overview of the approach used to calculate the database of phase field simulations used in this work.

We validate the usefulness of the dataset by training two standard machine learning models, a U-Net and a SegFormer, on the dataset. The ML models are briefly introduced and the results of the evaluation are presented.

### Phase Field Governing Equations

#### Bulk equations

Presented phase field simulations are based on Cahn-Hilliard equation^23^. The Cahn-Hilliard equation is very common for prediction of microstructure evolution in a systems undergoing phase separation. The equation is as follows:

1 $$\frac{\partial c}{\partial t}=\nabla \cdot \left({\bf{M}}\nabla \mu (c)\right).$$

Here, the order parameter *c* represents the concentration field of the species driving phase separation (dependent variable), *t* denotes time (independent variable), **M** signifies the species’ mobility, and *μ*(*c*) is the chemical potential function of concentration. The solution to the partial differential equation (1) requires knowledge of the dependency *μ*(*c*). A common approach involves deriving *μ*(*c*) from the total free energy ${\mathcal{F}}$, expressed as a volume integral of the free energy density *f*(*c*(**r**, *t*)) and gradient penalty term $\frac{\kappa }{{c}_{m}^{2}}{[\nabla c({\bf{r}},t)]}^{2}$ penalizing phase boundaries in the system:

2 $${\mathcal{F}}={\int }_{V}\left[\,f(c({\bf{r}},t))+\frac{\kappa }{{c}_{m}^{2}}{[\nabla c({\bf{r}},t)]}^{2}\right]dV.$$

Here, *c*_*m*_ is the maximum concentration, and *κ* is the gradient penalty coefficient.

For effective description of phase separation, the free energy density *f*(*c*) must exhibit two local minima at different values of the phase field variable *c*. We chose the second-order parabolic form with two additional logarithmic terms (equation (3)), derivable from regular solution theory, following the original work by Cahn and Hilliard^23^.

3 $$f(c)=RTcln\left(\frac{c}{{c}_{m}}\right)+RT\left({c}_{m}-c\right)ln\left(\frac{{c}_{m}-c}{{c}_{m}}\right)+\Omega \frac{c({c}_{m}-c)}{{c}_{m}^{2}}+\frac{{B}_{0}}{2{c}_{m}^{2}}{(c-\bar{c})}^{2}.$$

Here, *R* is the gas constant, *T* is temperature, and *Ω* represents the regular solution parameter. The additional term $\frac{{B}_{0}}{2{c}_{m}^{2}}{(c-\bar{c})}^{2}$ in the regular solution free energy density accounts for the strain contribution of phase boundaries to the total system energy, where $\bar{c}$ is the average concentration.

Applying the Lagrange minimization principle to the total free energy functional ${\mathcal{F}}$ (equation (2)) yields the chemical potential *μ*(*c*), which equilibrates across the system at thermodynamic equilibrium:

4 $$\mu (c({\bf{r}},t))=RT\,ln\frac{c({\bf{r}},t)}{{c}_{m}-c({\bf{r}},t)}+\Omega \left(1-\frac{2c({\bf{r}},t)}{{c}_{m}}\right)-\nabla {\boldsymbol{\kappa }}\nabla c({\bf{r}},t)+{B}_{0}\left[\frac{c({\bf{r}},t)-\bar{c}(t)}{{c}_{m}^{2}}\right].$$

Equations (1) and (4) together form a solvable system used for phase field simulations of Cahn-Hilliard systems.

#### Parametrization

The Cahn-Hilliard equation system described in section generally involves fifteen parameters (note that **M** and **κ** are tensors with six components in their most general form). To solve the equation system, we adopt a parameterization calibrated for describing the concentration field within the active particles of lithium iron phosphate (LiFePO_4_) in Li-ion battery electrodes^17,20^. In this specific case, detailed in^20^, the number of parameters in Equations (1) and (4) reduces to five, owing to the two-dimensional nature of the described particles and their planar isotropy of **M** and **κ**. Mobility of diffusing species was replaced by relation ${\bf{M}}=\frac{Dc}{RT}$ where *D* is diffusion coefficient. Material parameter values (*D*, **κ,** *c*_*m*_, *Ω* and *B*_0_) for LiFePO_4_ were obtained from the literature and are presented in Table 1.

**Table 1:** Material parameters used in phase field model of LiFePO_4_ nano-particle.

Parameter	Value	Units	Reference
*D*	8 × 10^−12^	cm^2^/s	^44^
**κ**	2.453 × 10^9^	eV/m	^45^
*c*_*m*_	22800	mol/m^3^	^45^
*Ω*	0.115	eV	^45^
*B*_0_	0.19	GPa	^44^

Utilizing parameterization for characterizing the LFP active electrode material, presented equations were solved within a two-dimensional rectangular domain, incorporating periodic boundary conditions.

### Numerical approach

The system of coupled partial differential equations, presented in section, is rewritten to the system of differential algebraic equations (DAE) using the finite volume method (FVM) on the two dimensional rectangular domain (2D) in the plane perpendicular to the (010) crystallographic direction of the material^30^.

The FVM was chosen due to its inherent conservation properties when applied to the equations containing divergence operators^31^, its straightforward implementation and its applicability to complex geometries. In the analysed case of a rectangular 2D domain, other numerical methods, such as spectral methods or implicit finite element methods, can also be used. Continuous boundary conditions are applied at the edges of the domain to simulate a section of the bulk active material. The modelling framework calculates the right hand side (RHS) and corresponding Jacobian terms based on the system of differential algebraic equations (DAE). DAE system is solved numerically using the Implicit Differential-Algebraic solver (IDA), a general purpose solver for the initial value problem (IVP) for systems of DAEs from the SUNDIALS software suite^32,33^. The code was tested and run with SUNDIALS library version 5.7.0 and compiled with the gcc compiler on the Linux operating system.

Various settings are available for the model in the first section of the code. Users can choose between a direct dense linear solver using a user-supplied Jacobian or a number of Krylov-based linear solvers^34^ (e.g. SPGMR, SPFGMR, SPBCGS) that use the diagonal elements of the Jacobian for preconditioning. In addition, the relative and absolute tolerances of the state variables can be varied depending on the use case. Another selection that the user can make is the choice of input current mode for running the model, i.e. direct current (DC) mode or alternating current (AC) mode. For the results in this paper, the DC mode was chosen with zero current applied to the computational domain. With the AC mode, for example, the galvanostatic EIS analysis can be performed at different excitation frequencies, resulting in Nyquist plots^20^. Several model parameters can also be varied (see Table 1) where all input parameters are specified in dimensional units, while the governing equations are implemented in a non-dimensional formulation for enhanced robustness and stability of the model.

### Creating a database of phase field trajectories

**Fig. 2:**
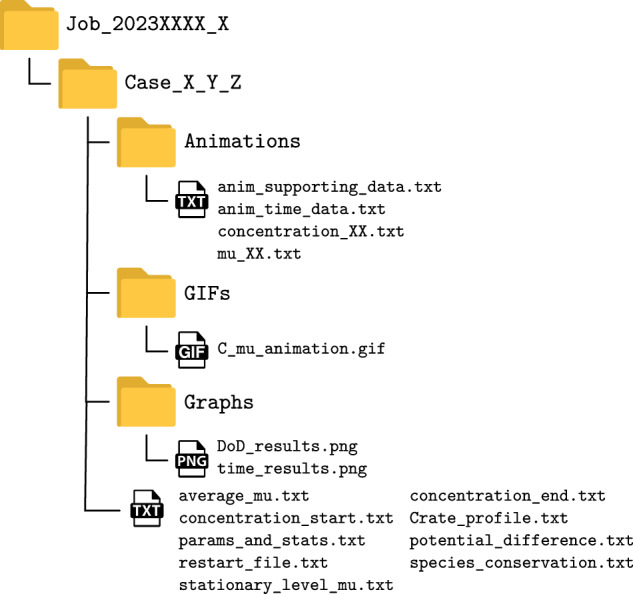
A schematic representation of the folder structure and the corresponding .txt output files of the phase field model and the post-processing images and animations from the Gnuplot script.

To create large data sets, a workflow must be used to automatically run and save the results of multiple simulation trajectories by varying the model parameters. The first step is to execute the compiled code of the model with command line arguments that specify certain parameters of the model to be varied (if no arguments are specified, the program uses default parameters). A supporting SLURM script has been written for performing large parametric study of the design space on the HPC architecture. The user defines arrays of values for each parameter to be varied. The script then distributes compiled executable across the computational node on the designated HPC with the for-loops for each combination of input parameters, i.e. full factorial. Each run of the script first creates a “Job” folder in the results that contains the date and ID number in the name. In addition, each parameter combination is labelled as a “Case” and for each case a designated folder is created within the “Job” folder with a name containing the values of the parameters separated by an underscore. Specifically for the database presented in this paper, the following parameters were varied: domain size, mean value of concentration and number of initial random distributions of concentration in the domain. For example the folder name “Case_11400_228_120e-9_3” corresponds to Case_[average concentration in mol/m^3^]_[initial random perturbation in mol/m^3^]_[domain size in meters]_[id number of initial random perturbation]. During the simulation, the model exports various results in a human-readable .txt format with the export frequency specified by the user. Detailed representation of the folder structure and description of output results is represented in Fig. 2 and Tables 3 and 4. If the steady-state was not reached in the selected number of time steps, the user can restart the simulations from the last saved state.

### Baseline evaluation with U-Nets and Segformers

The dataset consists of 1100 simulation trajectories, 110 trajectories in each domain size. We first separated all trajectories with a domain size of 160 nm, consisting of 110 trajectories. Those trajectories were not used for training or testing. From the remaining trajectories, 132 were randomly allocated to test data that is not used during training but only to evaluate the performance afterwards. We evaluated the performance on the dataset with two popular machine learning architectures for segmentation, U-Nets and Segformers^35,36^. U-nets were introduced in 2015 by Ronneberger *et al*.^35^ as a convolutional neural network specifically crafted for image segmentation tasks. It is comprised of a contracting path for hierarchical feature extraction and an expansive path for high-resolution localization. Noteworthy are the skip connections that concatenate feature maps from the contracting path to the expanding path, preserving fine-grained spatial information. The incorporation of convolutional and pooling layers in the encoder allows efficient context capturing, while the decoder employs transposed convolutions for precise localization. U-Nets are very data-efficient and can en- and decode local as well as global features. To avoid the risk of overfitting and to maintain simplicity in the transformation process between steps, we intentionally selected the original U-Net architecture over more complex alternatives, such as the nested U-Net.

To compare to another popular architecture we also compare against SegFormers in some experiments^36^. SegFormers are a transformer-based light-weight architecture^37^ comprised of an encoder with multiple transformer blocks and a decoder.

All training was done in PyTorch on a Nvidia RTX 3090 GPU^38^. We used the AdamW optimizer for training with a standard initial learning rate of 1*e* − 4^39^. If the validation loss had not improved for three concurrent epochs, the learning rate was reduced to 10% of the previous learning rate. The training was continued until the validation loss had no longer improved for twenty concurrent epochs. Due to the relatively large size of the data set, one epoch is defined as 60.000 samples for training and 20.000 samples for validation to allow more fine-grained insights into how fast the ML model converges. After initial experiments with MSE (Mean Square Error) and BCE (Binary-Cross-Entropy) we ran subsequent experiments with MSE as the loss function since it showed a slight improvement over BCE.

## Data records

The data can be downloaded at Rieger *et al*.^40^ and the raw data can be downloaded at Rieger *et al*.^41^.

Table 2 represents the DoE matrix of the raw dataset with 10 domain sizes, 11 average Li concentrations within the domain and 10 initial random distributions of concentration, resulting in 1100 different cases. The data are organised so that each top-level folder contains 110 cases from a single domain size. Table 3 contains descriptions of the individual folders that are contained inside the top-level Job folder as shown in Fig. 2, whereas the Table 4 contains descriptions of each individual file that is generated either by the phase field model itself or the Gnuplot script for the post-processing visual analysis, i.e. plots and animations.

**Table 2:** DoE matrix of the dataset.

Domain size	Average concentration	Number of initial
(nm × nm)	(mol/m^3^)	random conc. distributions (-)
30 × 30	5700	10
40 × 40	6840	
60 × 60	7980	
80 × 80	9120	
100 × 100	10260	
120 × 120	11400	
140 × 140	12540	
160 × 160	13680	
180 × 180	14820	
200 × 200	15960	
	17100

**Table 3:** Description of the folder structure schematically presented in Fig. 2.

Folder name	Description
Job_2023XXXX_XX	Top level folder containing different Cases within the Job
Case_X_Y_Z	Case folder with simulation results with a chosen variation of parameters
Animation	Folder containing concentration and chemical potential results for each frame
GIFs	Folder containing GIF animation of the spinodal decomposition of the simulation
Graphs	Folder containing time and DoD-resolved results

**Table 4:** Description of the output files of the phase field model schematically presented in Fig. 2.

File name	Description	Columns description
anim_supporting_data.txt	Supporting data for gnuplot animation script	/
anim_time_data.txt	Data containing information for each frame of the animation	Frame index [-]; Time stamp of the frame [s]; Non-dimensional C-rate [-]; Depth of discharge [-]; Average chemical potential [-]
concentration_XX.txt	Non-dimensional concentration values in the 2D domain	Normalised x-coordinate [-]; Normalised y-coordinate [-]; Non-dimensional concentration [-]
mu_XX.txt	Non-dimensional chemical potential values in the 2D domain	Normalised x-coordinate [-]; Normalised y-coordinate [-]; Non-dimensional chemical potential [-]
C_mu_animation.gif	GIF animation of the spinodal decomposition of the simulation	/
DoD_results.png	Plot of the DoD-resolved results	/
time_results.png	Plot of the time-resolved results	/
average_mu.txt	Time resolved non-dimensional average chemical potential	Time [s]; Average non-dimensional chemical potential [-]
concentration_end.txt	2D matrix of the non-dimensional concentration field at the end of simulation	/
concentration_start.txt	2D matrix of the non-dimensional concentration field at the start of simulation	/
Crate_profile.txt	Time resolved non-dimensional C-rate	Time [s]; Non-dimensional C-rate[-]
params_and_stats.txt	Parameters and statistics of the simulation run	
potential_difference.txt	Time resolved potential difference	Time [s]; Non-dimensional potential difference [-]
restart_file.txt	File including data for restarting the simulation	/
species_conservation.txt	Time resolved species conservation	Time [s]; Non-dimensional average concentration in the domain [-]
stationary_level_mu.txt	Time resolved value of stationary level of non-dimensional chemical potential	Time [s]; Non-dimensional value of stationary level of chemical potential [-]

The data consists of 1100 trajectories. For ML training, we have first set aside 160 nm to serve as a dataset to test the OOD (out-of-distribution) performance on. During training and validation the model will not see any trajectories of this domain size, allowing us to measure how well the model generalizes to a previously unseen case. For the remaining trajectories we have randomly split the trajectories into a training, validation and test dataset with a 70:15:15 split. Due to a high amount of information overlap within the trajectories, e.g. the prediction from the 1st to the 50th and the 2nd to 51st being similar, we split the data by trajectory to avoid unintentional data leakage.

To utilize the dataset in a machine learning (ML) project, the data is available as multiple HDF5 files, consisting respectively of the training/validation/test/out-of-distribution data. To use the dataset for training a ML model, the data can easily be loaded into Python. We provide a custom PyTorch dataset class for this purpose. We refer to the published code for an example of how the data can be loaded and used to train an ML algorithm.

The processed data is available at Rieger *et al*.^40^. The raw data is available at Rieger *et al*.^41^

## Technical validation

We evaluate the performance of a strong baseline, the U-Net architecture against the dataset. In addition we also compare against a recently introduced ML algorithm, SegFormers, a transformer based approach for segmentation. For all experiments we report the performance as the error of the end points for each trajectory in the text set. The start point, i.e. the last computed image the ML model is shown is taken as a hyperparameter and varies. The error is computed in the two-class case as $er{r}_{abs}=\frac{1}{N}{\sum }_{n=1\ldots N}| {y}_{pred,n}-{\widehat{y}}_{n}|$

with *N* being the total number of trajectories in the test set, ${y}_{pred,n}\in {{\mathbb{R}}}^{100\times 100}$ being the final phase field of the *n*_*th*_ trajectory as predicted by the ML model and ${\widehat{y}}_{n}\in {{\mathbb{R}}}^{100\times 100}$ being the computed final phase field of the *n*_*th*_ trajectory.

The error varies between 0 (perfect prediction) and 1 (no overlap between the predicted and computed phase field). In addition to the quantitative evaluation we show a trajectory from the test set as predicted by the U-Net in Fig. 5. The test set trajectories were randomly taken from the entire dataset. To additionally examine whether the ML model can correctly predict the trajectory for a domain size it has not seen during training we have before the train/val/test split allocated all trajectories from the 160 nm domain size as an additional data set. The ML model was not trained on trajectories with this domain size. We report the performance on this dataset (referred to as ’OOD error’) in Fig. 6, showing that the error for this domain size is consistent with the errors for 140 nm and 180 nm. This indicates that the architecture can interpolate to the previously unseen domain size.

For training the trajectories are converted into pairs such that the U-Net is trained to predict the phase field at next step from the current step. For testing, unless otherwise noted, the error is computed by inputting the starting phase field into the U-Net and iteratively predicting the next step followed by computing the difference between the final prediction and computed phase field.

**Fig. 3:**
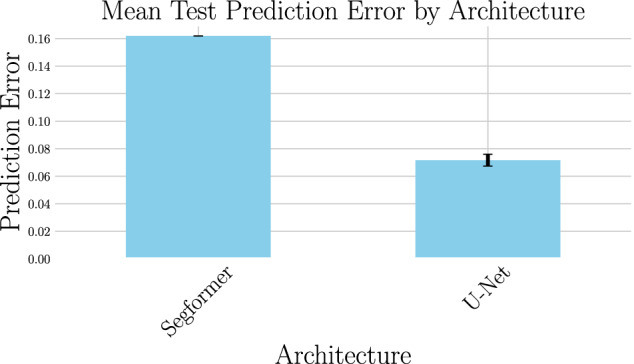
Comparing the performance of U-Net and SegFormer for a starting offset of 10 and a prediction offset of 10.

**Fig. 4:**
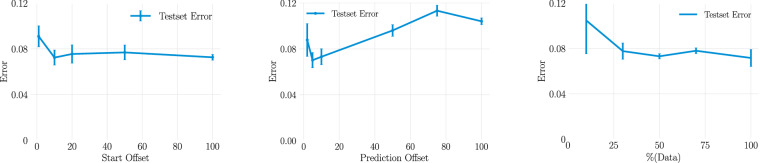
Visualizing the impact of the starting offset, prediction offset and size of the dataset on the predictive performance of the U-Net. If not otherwise specified, the starting offset is fixed at 10, 100% of the data is used and the prediction offset is fixed at 5. On the left we visualize the error dependent on starting offset. In the middle we visualize the error dependent on prediction offset. On the right we visualize the error dependent on the training data size as a percentage of the entire dataset.

**Fig. 5:**
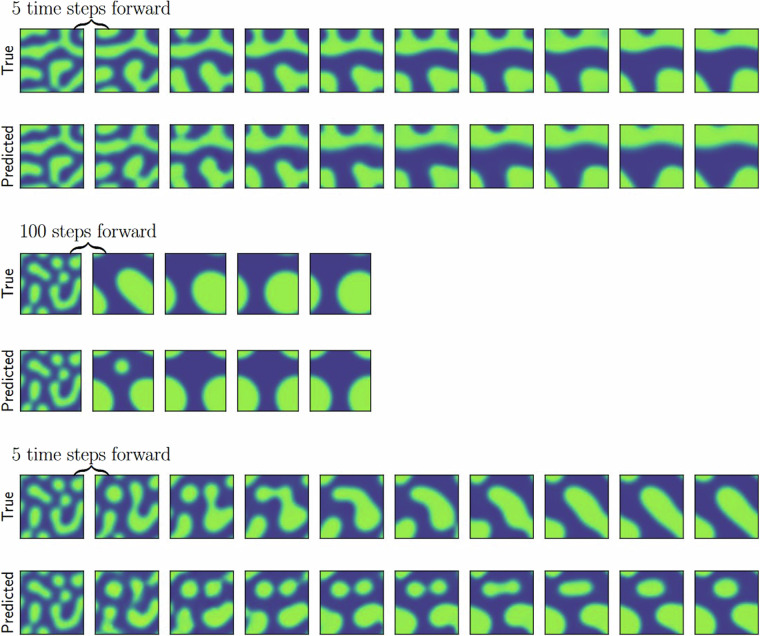
We show example trajectories from the test set as predicted by the machine learning model (bottom row in each simulation) and obtained by the computational simulation (top row in each simulation). Since the development is rapid in the beginning and slows down we show the development in two different resolutions, every fifth simulation step in the upper row and every hundredth simulation step in the middle row. The lower row shows an example where the predicted trajectory is not correct.

As shown in Fig. 3 the standard U-net architecture has a lower prediction error than the SegFormer when predicting entire trajectories. In the right column of Fig. 4 we show the absolute error when the size of the training dataset is varied (shown as the percentage of the full training dataset). The starting offset, the number of simulation steps that were not taken into account in the beginning, is fixed at 10 and the prediction offset is fixed at 5. The ML model improves with more data until 50% of the dataset at which point the improvement levels off, indicating that adding more data is no longer advantageous for the model’s accuracy. In the left column of Fig. 4 we show the impact of varying the amount of steps taken by the computational simulation before the trajectory is predicted by the U-Net. As expected, the error is reduced when the prediction is started later as the high energy in the earlier stages as seen in Fig. 5 are harder to predict.

In the middle column of Fig. 4 the prediction offset, the number of steps the ML model ’jumps’ forward in each prediction step, is varied. This parameter signifies the amount of simulation steps the ML model predicts forward in time in each step. With a small number the amount of inferring per step is reduced but the error accumulated in each step is increased as more predictions are necessary. As such, the curve displays a U-shape with the lowest error at a step size of 5.

**Fig. 6:**
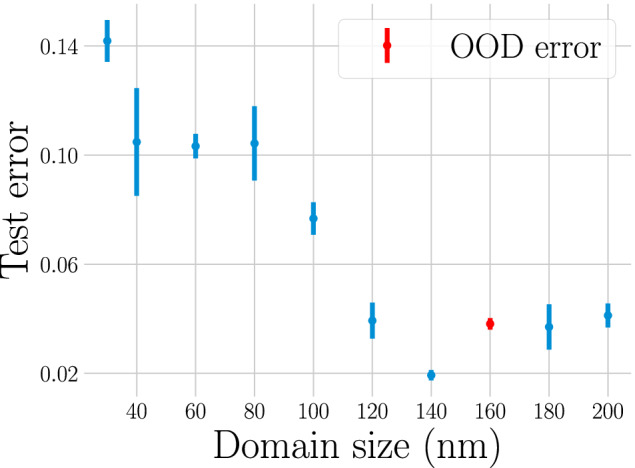
Error on data from the test set dependent on the domain size. Despite all domain sizes being equally present in the training data the error is strongly inversely related to the domain size. The error for a domain size of 160 nm (not seen during training) is consistent with other domain sizes indicating that the U-Net can generalize to the domain size.

To evaluate the influence of the domain size on the network performance, we visualize the error dependent on the domain size in Fig. 6. The error varies strongly with the domain size from below 0.03 for a domain size of 180 nm to  ≈ 0.17 that at 30 nm, underlining the need for a standardized dataset such as we present here. An ostensibly new state-of-the-art can easily be reached just by choosing a different domain size for the dataset. We further note that the error curve seems to approximate a U-shape with a slight trend to increase again above 180 nm. Since all domain sizes are roughly equally present in the dataset, it is unlikely that the error is due to having overfit to a particular domain size. Additionally, our dataset allows for the development of domain-size independent ML algorithms as the ML model learns to generalize over multiple domain sizes.

To summarize, we present a novel dataset consisting of a wide range of domain sizes. We show that a standard U-Net with a novel self-consistency enforced can reach good predictive accuracy on this task when evaluated on predicting entire trajectories. While it is not possible to compare directly to previous work, since the performance on different datasets can vary widely as demonstrated in Fig. 6, the results are within range of previous work, e.g  ≈ 2% error indicated in Oommen *et al*.^42^ and 2% error for a domain size of 140nm as seen in Fig. 6.

To be able to compare against e.g.^24^ we also compute the error of the ARE (Absolute relative error) of the average feature size for the final frames of the test set for one model. The ARE is defined as in de Oca Zapiain *et al*.^24^ as the absolute difference between the average feature size as predicted and computed, divided by the computed average feature size. The average feature size is computed as the first minimum of the radial average of the microstructure’s autocorrelation.

With an ARE of 2.6% for the U-Net with a starting offset of 10 and a prediction offset of 5 our approach compares favorably against e.g. de Oca Zapiain *et al*.^24^, Hu *et al*.^43^ with an ARE of 5% though it should again be stressed that the results are not directly comparable since the datasets are different.

The lack of comparability highlights the need for a standard open-source and easily usable dataset as we present here.

## Data Availability

All code needed to recreate the results presented here and to use the benchmark dataset is published under https://github.com/laura-rieger/phase_field_benchmark. The code is written in Python using the PyTorch library^38^. All scripts and notebooks necessary to reproduce the results in this paper can be found in the Github repository.
